# Supplementary material for: Oncotype DX Test Receipt among Latina/Hispanic Women with Early Invasive Breast Cancer in New Jersey: A Registry-Based Study
Source: Int J Environ Res Public Health. 2021 May 12;18(10):5116. doi: 10.3390/ijerph18105116 (PMC8151910; doi:10.3390/ijerph18105116)
Supplement: Supplementary file 1 [file ijerph-18-05116-s001.zip › ijerph-1190213-SI.pdf]

International Journal of
Environmental Research and Public Health

MDPI

Supplementary Material:

Table S1. Sociodemographic characteristics of Latina/Hispanic women diagnosed with invasive breast cancer in New Jersey, 2008–2017 (N = 5777).

**Variables**	***n* (%)**
Age at Diagnosis [a]	56.54 ± 13.51
Year of Diagnosis	
2008	455 (7.9)
2009	482 (8.3)
2010	465 (8.0)
2011	491 (8.5)
2012	554 (9.6)
2013	590 (10.2)
2014	624 (10.8)
2015	693 (12.0)
2016	715 (12.4)
2017	708 (12.3)
Race	
White	5183 (89.7)
Black	277 (4.8)
Other (American Indian/AK Native, Asian/Pacific Islander)	284 (4.9)
Missing/unknown	33 (0.6)
Ethnic Subgroup [b]	
Mexican	215 (3.7)
Puerto Rican	1010 (17.5)
Cuban	322 (5.6)
Dominican	345 (6.0)
South or Central American	1229 (21.3)
Spanish/Hispanic/Latino NOS	1642 (28.4)
Other	1014 (17.5)
Nativity	
U.S. Born	733 (12.7)
Non-US-born	2367 (40.9)
Missing/unknown	2680 (46.4)
Insurance Status	
Uninsured	618 (10.7)
Medicaid	901 (15.6)
Insured	3201 (55.4)
Insured, but not specified	729 (12.6)
Missing/unknown	328 (5.7)
Area-Based Composite Socioeconomic Status [c]	
Low	1199 (20.7)
Low-middle	1183 (20.5)
Middle	1028 (17.8)
Middle-high	1152 (19.9)
High	1154 (20.0)
Missing/unknown	61 (1.1)

[a] Age at Diagnosis given as mean ± standard deviation. [b] Ethnic Subgroup was recoded using the Indirect Identification from the NAACCR Hispanic/Latino Identification Algorithm [NHIA v.2.2.1] of "Surname match only" (sensitivity = 84.37% & specificity = 99.14%). [c] Area-Based Composite Socioeconomic Status was based on the Yost Index using US 2010 Census Tract data.
